# LOCI RESPONSIBLE FOR RACIAL DISPARITY BETWEEN WHITE AND BLACK AMERICANS IN ALZHEIMER’S DISEASE

**DOI:** 10.1093/geroni/igac059.2210

**Published:** 2022-12-20

**Authors:** Stanislav Kolpakov Nikitin, Igor Akushevich

**Affiliations:** Duke University, Durham, North Carolina, United States; Duke University, Durham, North Carolina, United States

## Abstract

We tested the following genotype-related mechanisms generating race-dependent disparity in Alzheimer’s Disease (AD) risk: i) the frequencies of SNPs with genotypes associated with AD are higher in the Black subpopulation; ii) the effects on AD risk of genotypes associated with increased AD risk is higher in the Black subpopulation; iii) there is a small group of SNPs with a large difference in the effects on AD race-dependent risk generating disparities; alternatively, the disparities are generated by a collective effect of multiple SNPs with minor or moderate effects. We modified the GWAS algorithm to use it with the Cox regression multivariable model and the outcome accounting for race-related differences in AD risk. Using the modified GWAS we have identified loci in charge for the disparity between Whites and Blacks and used the results along with SNPs of a special interest which were identified from the literature in the Cox multivariable regression model and generalized Oaxaca-Blinder approach. The following genes had the strongest effects on racial disparities: i) NYAP1 neuronal tyrosine-phosphorylated phosphoinositide-3-kinase adaptor; ii) RPA3-UMAD1 loci RPA3 replicates protein A, interacting with gastric tumor and hepatocellular carcinoma; iii) TOMM40 associated with an increased risk of developing late-onset AD; iv) ACE - responsible for making the enzyme which converts angiotensin which regulates blood pressure; v) PSME3IP1 - proteasome activator subunit 3 interacting protein 1; vi) MARCHF1 regulator of glucose-tolerance and lipid storage and vii) LINC01146 which belongs to coagulation cascade pathway and linked to hepatocellular carcinoma.

